# Identification of Digital Health Priorities for Palliative Care Research: Modified Delphi Study

**DOI:** 10.2196/32075

**Published:** 2022-03-21

**Authors:** Amara Callistus Nwosu, Tamsin McGlinchey, Justin Sanders, Sarah Stanley, Jennifer Palfrey, Patrick Lubbers, Laura Chapman, Anne Finucane, Stephen Mason

**Affiliations:** 1 Lancaster Medical School Lancaster University Lancaster United Kingdom; 2 Marie Curie Hospice Liverpool Liverpool United Kingdom; 3 Liverpool University Hospitals National Health Service Foundation Trust Liverpool United Kingdom; 4 Palliative Care Unit University of Liverpool Liverpool United Kingdom; 5 Dana-Farber Cancer Institute Boston, MA United States; 6 Ariadne Labs Brigham and Women's Hospital and Harvard T H Chan School of Public Health Boston, MA United States; 7 Harvard Medical School Harvard University Boston, MA United States; 8 Princess Alice Hospice Surrey United Kingdom; 9 Netherlands Comprehensive Cancer Organization (IKNL) Utrecht Netherlands; 10 Clinical Psychology, University of Edinburgh Edinburgh United Kingdom

**Keywords:** palliative care, terminal care, supportive care, quality of life, symptom management, digital health, technology

## Abstract

**Background:**

Developments in digital health have the potential to transform the delivery of health and social care to help citizens manage their health. Currently, there is a lack of consensus about digital health research priorities in palliative care and a lack of theories about how these technologies might improve care outcomes. Therefore, it is important for health care leaders to identify innovations to ensure that an increasingly frail population has appropriate access to palliative care services. Consequently, it is important to articulate research priorities as the first step in determining how finite resources should be allocated to a field saturated with rapidly developing innovation.

**Objective:**

The aim of this study is to identify research priority areas for digital health in palliative care.

**Methods:**

We selected digital health trends, most relevant to palliative care, from a list of emerging trends reported by a leading institute of quantitative futurists. We conducted 2 rounds of the Delphi questionnaire, followed by a consensus meeting and public engagement workshop to establish a final consensus on research priorities for digital technology in palliative care. We used the views of public representatives to gain their perspectives on the agreed priorities.

**Results:**

A total of 103 experts (representing 11 countries) participated in the first Delphi round. Of the 103 experts, 55 (53.3%) participated in the second round. The final consensus meetings were attended by 10.7% (11/103) of the experts. We identified 16 priority areas, which involved many applications of technologies, including care for patients and caregivers, self-management and reporting of diseases, education and training, communication, care coordination, and research methodology. We summarized the priority areas into eight topics: big data, mobile devices, telehealth and telemedicine, virtual reality, artificial intelligence, smart home, biotechnology, and digital legacy.

**Conclusions:**

The priorities identified in this study represent a wide range of important emerging areas in the fields of digital health, personalized medicine, and data science. Human-centered design and robust governance systems should be considered in future research. It is important that the risks of using these technologies in palliative care are properly addressed to ensure that these tools are used meaningfully, wisely, and safely and do not cause unintentional harm.

## Introduction

### Background

Developments in digital health (describing technologies that use computing platforms, connectivity, software, and sensors for health care and related purposes) have the potential to transform the delivery of health and social care to help citizens manage their own health [1-3]. Currently, there is a lack of consensus about digital health research priorities in palliative care and theories about how these technologies might improve care outcomes. Therefore, it is important to articulate research priorities as the first step in determining how finite resources should be allocated to a field saturated with rapidly developing innovation. Global palliative care needs are expected to increase because of the consequences of an aging population; therefore, it is important for health care leaders to identify innovations to ensure that an increasingly frail population has appropriate access to palliative care services [4]. Research demonstrates that, when used well, digital health initiatives improve health care delivery and access [5-15], and the World Health Organization suggests that digital health should be an integral part of health priorities as a means to improve health on a global scale [16,17]. To date, many barriers have prevented the meaningful use of digital health in palliative care [18], including expenses, interoperability issues, data privacy and security concerns, lack of effectiveness and equity, and the concern that technology will reduce face-to-face consultations between patients and clinicians [19,20].

Strategic forethought (futurism) can help palliative care leaders recognize emerging trends and test, plan, and use these innovations in practice [21]. Consequently, this study aims to identify digital health research priorities and to theorize how innovations in emerging technologies can improve palliative care.

### Aim

The aim of this study is to identify research priority areas for technology in palliative care.

## Methods

### Study Design

We used a Delphi process, informed by the Guidance on Conducting and Reporting Delphi Studies [22] in palliative care, to establish the opinions of palliative care experts. A Delphi process can be used as a consensus-based, forecasting process, enabling anonymous expert contributions to predict phenomena [23,24]. We chose to use the Delphi method because of its potential to achieve consensus in areas of uncertainty [25-28]. We conducted 2 rounds of the Delphi questionnaire, followed by a consensus meeting and public engagement workshop to establish a final consensus on research priorities for digital technology in palliative care. Data were collected between November 2018 and September 2019.

### Identification of Technology Trends From the Future Today Institute

We selected technology trends most relevant to palliative care from a list of emerging technology trends reported by the Future Today Institute (FTI) [29]. The FTI is a multi-professional organization that uses data-driven applied research to develop models that forecast risks and opportunities across several disciplines, which are mapped into technology trends. The 2018 trend list included 225 emerging trends, which were stratified by FTI authors into 19 categories (Multimedia Appendix 1).

### Selection of Technology Trends for Palliative Care

**Figure 1:**
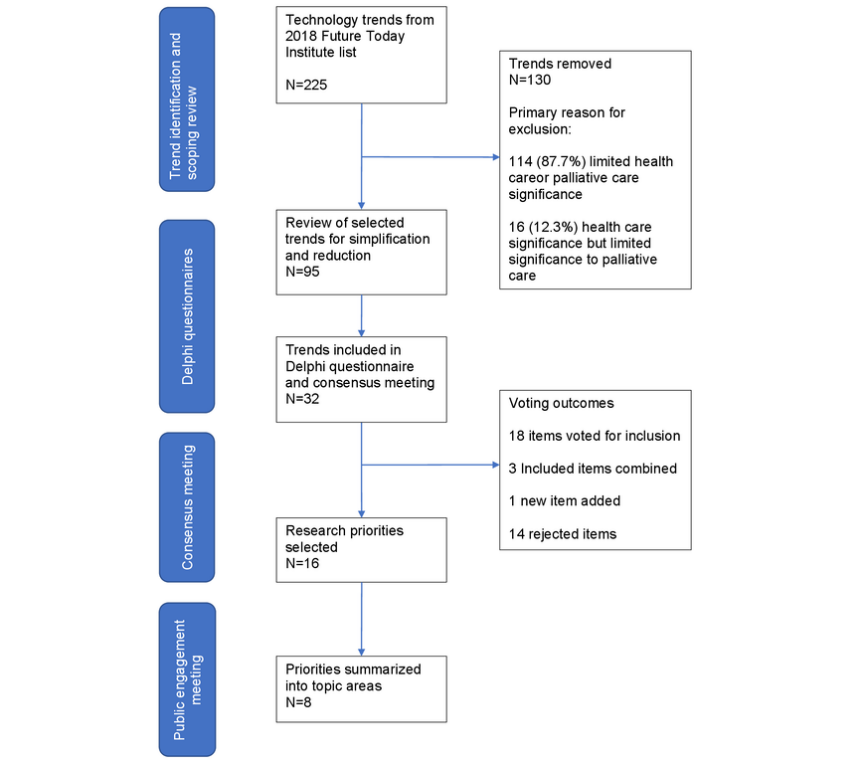
Flow diagram to outline study process for identifying research priority areas.

We developed criteria to select the FTI trends based on recommendations from a UK-based policy report, which reported public and professional views on new types of health care data [30]. We developed the following statement to select FTI trends for inclusion: “Trends should involve analysis or use data generated by a patient, caregiver or healthcare professional with potential use in palliative care.” A total of two authors (ACN and TMcG) reviewed all 225 FTI trends. We chose to review all FTI trends (despite their previous categorization) to ensure that no suitable trends, from categories deemed less relevant to palliative care (eg, agricultural technologies, space, and government and technology policy), were overlooked. We included 42.2.% (95/225) of the trends. We then combined and simplified similar trends to reduce the number to 32 (32/225, 14.2%; Figure 1). To confirm the validity of the trends in palliative care, we conducted a focused literature review to identify examples in which these technologies had been used in health care. An Excel (Microsoft Inc) spreadsheet was used to collate the data for reference.

### Delphi Questionnaire Development

We developed 32 items for inclusion in the Delphi questionnaire, which reflected the 32 trends identified in the FTI Report (Figure 1). We used Google Forms (Google Inc) [31] to develop the survey. We designed a questionnaire to collect demographic information (geographic location, age, and occupation) and individuals’ rating of importance for each item using a 5-point Likert scale (1=low priority to 5=high priority). To ensure that the survey questions were appropriate, we conducted a local prestudy pilot of the questionnaire and supporting materials (Multimedia Appendices 2 and 3).

### Participant Recruitment and Consent

We solicited a convenience sample of professionals working in palliative care (including physicians, nurses, social workers, therapists, pharmacists, spiritual care staff, and managers) who were interested in technological innovation. We used professional networks, social media, and email to contact individuals (Multimedia Appendix 4). Consenting participants accessed the study material on the internet to complete an electronic consent form and a first-round Delphi questionnaire. Participants who completed the first round of the questionnaire were invited to participate in the second round.

### Ethical Approval

This study was approved by the University of Liverpool Ethics Committee (approval number 3564).

### Data Collection and Analysis

Quantitative statistical analyses of participant ratings were performed using the statistical software package SPSS (version 22.0; IBM Corp). We used the IQR to determine the level of agreement on the 5-point scales for each *area* on the questionnaire. The justification for the levels of agreement was based on thresholds previously used in palliative care Delphi studies, which used a 5-point Likert scale to determine agreement (Multimedia Appendix 5) [22,32]. We emailed a summary of the first-round Delphi results to each participant. The email included the following information: (1) a summary of how the participant rated each item in the first Delphi round and (2) a summary of all participants’ responses for each item (pooled level of agreement). We provided this information so that participants could consider whether they wished to rank items differently in the second Delphi round, based on the ranking data generated by other participants.

### Round 2 Delphi Questionnaire

We provided the participants with an electronic link to access the second-round Delphi questionnaire. We asked the participants to answer the same questions that were included in the first-round questionnaire. Participants were required to complete the questionnaire within 4 weeks. We analyzed the responses from the second questionnaire by IQR to provide a final list of items according to their level of agreement.

### Final Consensus Meeting and Voting

We organized a consensus meeting to agree with the trend list as the final stage of the Delphi process [22]. All participants were invited to attend a meeting at the University of Liverpool, United Kingdom. The participants were divided into 2 groups. We attempted to ensure the groups were similar by allocating individuals according to gender, experience, and occupation. We provided participants with the Delphi results via (1) an oral presentation and (2) a written summary. ACN and TMcG acted as group facilitators, and ACN chaired the meeting. We facilitated the group discussions and voting. Each item was discussed and debated, and a *raised-hand* vote was undertaken within each group to determine whether each item was included or excluded from the final list.

After voting, we compared the outcomes between the 2 groups. Items were included if both groups voted for inclusion. Similarly, items were excluded if both groups voted for exclusion. When the groups disagreed (ie, one group voting for inclusion and the other voting for exclusion), we facilitated debate with both groups together, which was followed by rounds of voting until consensus was achieved.

### Public Engagement Workshop

Following the consensus meeting, we conducted a public engagement workshop with lay representatives to determine their views on agreed priorities. Volunteer coordinators from the Marie Curie Hospice Liverpool and Liverpool University Hospitals National Health Service Foundation Trust invited palliative care volunteers (by telephone and email).

## Results

### Round 1 Delphi Questionnaire

Round 1 included 103 participants (Table 1). The median age of participants was 45 (SD 11.2) years. Most participants were women (65/103, 63.1%) and had a clinical background (74/103, 71.9%). The participants represented 11 countries, most commonly the United Kingdom (88/103, 85.4%). Most trend items (25/32, 78%) achieved a median priority rating of 4 or 5 (Multimedia Appendix 6), which suggested that participants considered most items to be important.

**Table 1:** Demographics of study participants (N=103).

Characteristics		First round (n=103)	Second round (n=55)	Consensus meeting (n=11)
Age (years), median (SD; range)		45 (11.2; 22-74)	44 (11.6; 22-74)	47 (11.5; 29-62)
**Gender, n (%)**				
	Male	38 (36.9)	23 (41.8)	4 (36.3)
	Female	65 (63.1)	32 (58.2)	7 (63.7)
**Location, n (%)**				
	United Kingdom	88 (85.4)	47 (85.5)	11 (100)
	United States	4 (3.9)	1 (1.8)	0 (0)
	Germany	2 (1.9)	2 (3.6)	0 (0)
	The Netherlands	2 (1.9)	1 (1.8)	0 (0)
	Saudi Arabia	1 (1)	0 (0)	0 (0)
	Canada	1 (1)	1 (1.8)	0 (0)
	Brazil	1 (1)	1 (1.8)	0 (0)
	Italy	1 (1)	0 (0)	0 (0)
	Sweden	1 (1)	0 (0)	0 (0)
	Argentina	1 (1)	1 (1.8)	0 (0)
	Austria	1 (1)	1 (1.8)	0 (0)
**Occupation, n (%)**				
	Clinical (nurse or physician)	74 (71.9)	38 (69.1)	6 (54.5)
	Academic	16 (15.6)	11 (20)	4 (36.4)
	Health care manager	4 (3.9)	2 (3.6)	1 (9.1)
	Layperson	3 (2.9)	3 (5.5)	0 (0)
	Allied health professional	2 (1.9)	1 (1.8)	0 (0)
	Chaplain	2 (1.9)	0 (0)	0 (0)
	Information technology	2 (1.9)	0 (0)	0 (0)

### Round 2 Delphi

Of the 103 participants in round 1, a total of 55 (53.3%) of the participants completed the round 2 questionnaire. The median age was 44 (SD 11.6) years, which was similar to that in round 1. More women than men completed the questionnaire (32/55, 58%). The distribution of occupations was similar across both the rounds. Fewer countries (8/11, 73%) were included in the final sample. The final IQR analysis (Multimedia Appendix 6) demonstrated that most items (21/32, 66%) had *low* levels of agreement, with 6% (2/32) and 28% (9/32) of the items achieving *moderate* and *high* levels of agreement, respectively.

### Consensus Meeting and Final List of Priorities

A total of 11 people participated in the consensus meeting (11/103, 10.7% of the total participants and 11/55, 20% of the second-round participants). The median age of the participants was 47 (SD 11.5) years, and most of them (7/11, 64%) were women. All participants were based in the United Kingdom and were mostly from clinical (6/11, 55%) or academic backgrounds (4/11, 36%). The debate resulted in agreement, rejection, modification (rewording and combination) of trends, and the addition of a new item, digital legacy (Multimedia Appendix 7). We classified the priorities into eight topic areas: big data, mobile devices, telehealth and telemedicine, virtual reality (VR), artificial intelligence (AI), smart home, biotechnology, and digital legacy (Multimedia Appendix 8).

### Public Engagement Event

We conducted a public engagement event at the Marie Curie Hospice Liverpool, United Kingdom, attended by 6 lay representatives, 2 staff members (nurse and physician), and a medical student. We began the meeting with a presentation discussing the importance of studying technology in palliative care. We then presented an overview of the Delphi outcomes, research topic areas, and identified priorities. We allocated attendees into 2 groups, and we (ACN and SS) facilitated 2 separate discussions (each lasting 45 minutes) with each group. Discussion 1 involved a discussion about the priorities from the big data, AI, and biotechnology topic areas. Discussion 2 involved discussion of priorities from telehealth and telemedicine, mobile devices and wearables, smart homes, VR, and digital legacy topic areas. We asked attendees for their views on priorities to determine their opinions on appropriateness and to identify areas that they believed warranted further study or clarification. Further information about the public engagement meeting is presented in Multimedia Appendix 9.

Our public representatives recommended that future research should (1) ensure a human-centered co-design approach to ensure that technologies are designed according to the needs of individuals and (2) appropriate governance processes should be in place to evaluate the efficacy, effectiveness, and ethical issues of current and future digital health tools and systems.

## Discussion

### Summary of Main Findings

This is the first study to identify digital health research priorities for palliative care and provide guidance for researchers, funders, and policy makers to consider areas for future research and development. We identified 16 priority areas, which involved many applications of technologies, including care for patients and caregivers, self-management and reporting of diseases, education and training, communication, care coordination, and research methodology. We summarized the priority areas into eight topics: big data, mobile devices, telehealth and telemedicine, VR, AI, smart home, biotechnology, and digital legacy.

### Contribution and Strengths of This Paper

#### Overview

The outcomes of our detailed analysis (involving a modified Delphi process and patient engagement workshop) indicate further digital health research is needed to study how technology can be best used to support palliative care. Our paper is the first priority-setting paper on palliative care digital health and provides a foundation for digital health–focused palliative care research.

#### Telehealth and Telemedicine

Before the COVID-19 pandemic, researchers highlighted the potential of using telehealth (ie, technology to support remote clinical access) and telemedicine (ie, technology to support remote clinical care delivery) in palliative care. These technologies are increasingly used in palliative care [33,34]; however, many have not been evaluated for use in real-world settings [19,35]. Beyond the pandemic, researchers can consider how these technologies can improve palliative care access (eg, for remote communities and hard-to-reach groups) to support new models of care (eg, telepalliative care clinics). It is also important to consider barriers (eg, equity of access, privacy, and security considerations), facilitators (ease of use and incentives), and use cases (eg, reasons for use) for the adoption of telehealth and telemedicine in palliative care.

#### Exploring AI

AI is often used as an umbrella term to describe several processes (eg, machine learning, natural language processing, deep learning, and neural networks) [36]. Clinicians and researchers are increasingly using AI to predict survival [37-40], classify pain severity [41,42], identify quality indicators [43,44], and identify serious illness conversations from electronic health care records [45]. However, most of these studies are exploratory and do not provide recommendations for clinical practice [18]. Therefore, researchers should explore how different AI techniques can support palliative care research and practice considering the ethical issues associated with these methods.

#### Big Data

Big data describes large amounts of (previously unmanageable) data that can now be processed by modern computer analysis techniques. The opportunities to use routine data to support palliative care decisions for populations and individuals have been reported previously [18,46]. Currently, there is no consensus on how nontraditional sources of big data can be meaningfully used in palliative care. For example, there is the potential to use patient-generated data (eg, wearables) for quality-of-life assessments. Furthermore, open-source genomic databases may provide opportunities to study the relationships between genetics and health to inform how data can be used for disease management. Social media and other forms of web-based data are increasingly used to support public and professional communication and to gain insight into public attitudes toward palliative care [47-50]. Consequently, researchers should identify which data to collect and how both traditional and nontraditional sources of palliative care big data can be best used [18,51,52].

#### Mobile Devices and Wearables

Many studies have described how mobile devices and wearables can support palliative care (eg, remote monitoring of physical activity and symptoms, delivery of well-being activities, documentation of advance care planning, education access or delivery, and guideline access) [53-57]. The capability of these devices to collect and store data is increasing; therefore, it is important to determine how meaningfully these data can be used [58,59]. Researchers have previously described how patient-reported outcomes can benefit palliative care patients [60-62]; however, further work is needed to explore how this technology can best support patient-reported outcomes collection (and use) in real-world settings [63,64]. It is important to examine how mobile devices are designed to meet the requirements of palliative care users [65]. Furthermore, studies should provide more information on how mobile devices can help patients record their care preferences (eg, advance care planning) [66,67].

#### VR Shows Great Potential for Palliative Care

VR is a human-computer interface technology that uses visual graphics, sounds, and other sensory inputs to create a web-based computer world [68]. Previous studies have described the potential of using VR to support psychosocial symptoms and well-being; however, most studies are unevaluated, so further research is needed [69-72]. We recognize the potential of VR to support palliative care education [73,74]; however, the consensus group did not identify this as a current priority. Following our study, we recognized that the COVID-19 pandemic has accelerated the use of web-based learning environments for medical education [75], particularly with the potential to use VR for communication skills training [73]. Consequently, it is possible that VR for education would be rated higher as a priority if this study were repeated.

#### The Smart Home

A smart home describes a living environment in which sensor-based systems and internet-connected devices (the internet of things) are used for remote monitoring and automation of appliances, such as lighting and heating [76]. Previous studies have illustrated how various technologies can support care for people experiencing a decline in their physical function (eg, web-based assistants and supportive robotics), which highlights the wider role these technologies may have in practice [77]. Consequently, future work should explore the usefulness of smart home technologies in supporting physical functions and the legal, privacy, and ethical issues associated with these developments [3,52,66,76-78].

#### Biotechnology

Biotechnology involves the combination of technologies with living things [79]. Palliative care–related developments include the use of biomarkers to predict survival [80,81], constipation, [82] and delirium [83,84], and the personalization of cancer pain according to genetics [85-87]. Consequently, it is possible to imagine future scenarios where technologies are used for early identification (and prediction) of clinical issues, facilitating personalized treatment for the individual (eg, early identification and management of pathological fractures).

#### Digital Legacy

A digital legacy is the digital information available about someone after death, such as social media, photos, videos, and gaming profiles [88]. The volume of digital information generated by citizens is increasing, creating new challenges after death [89]. The increasing use of cloud storage and social media contributes to uncertainty in data ownership, which creates difficulties for caregivers in managing the digital legacy of the deceased. Studies have demonstrated that health care professionals can positively support their patients in managing their digital legacy [88,90,91]. However, digital legacy is not routinely discussed in clinical practice, which means that we generally do not know how individuals want their data to be managed after death [92]. Therefore, we believe that researchers should explore how patients and caregivers can be supported to manage their digital legacy after death, with an exploration of the different methods and materials that can be used.

### Relation to Previous Work in This Area and Areas of Interest Following the Novel COVID-19 Pandemic

Our study is synergistic with previous work, which has been conducted across topic areas [19,35]. We acknowledge that our study predates the pandemic and it is possible that the priorities we identified may now have shifted. However, we believe that our research findings are valid, as the digital health innovations adopted during the pandemic are in sync with our priority list. (Multimedia Appendix 10 [47,50,93-113]) [34,35]. For example, telehealth was commonly used during the pandemic, with many palliative care services using it to provide remote clinical support [93-105], communication [106], and education [107]. Technologies have been used to maintain connections and to develop communities of palliative care practice [108,109]. VR is used to provide psychological care and symptom management [110,111]. In general, the findings of these studies describe the potential benefits of digital health; however, the rapid implementation of these technologies has created a number of challenges (eg, technical issues, data security, and well-being considerations) that require further evaluation [106]. We are encouraged that these palliative care digital health studies, conducted during the pandemic, are within the scope of our identified priorities. Evidence suggests that the pandemic has accelerated the adoption of digital health in palliative care practice (and related research in these areas), rather than shifted to different priorities to the ones we identified. We expect the development and evolution of digital health research areas, which may be new *priorities* or linked to existing areas; for example, AI-driven data analysis of data from *internet of things* devices. Consequently, we believe that the COVID-19 pandemic has elevated the importance of digital health, as health organizations use technology to support palliative care after the pandemic.

### Palliative Care Digital Health Priorities in Regions Unrepresented in This Study

Although geographic regions are unrepresented in our study (eg, Asia Pacific and Australasian or African regions), studies from these countries are consistent with our outcomes as they describe the emerging importance of palliative care digital health. Australian palliative care providers report digital health priorities that are similar to those identified in our study, with providers wanting innovations in the areas of client health records, telehealth, and personal health tracking [114]. However, digital health priorities are likely to differ between countries owing to geopolitical and socioeconomic drivers. For example, in Sub-Saharan African, digital health is not as established as in other high-income regions [115]. Consequently, Sub-Saharan African stakeholders describe digital health as part of a wider vision in this region to potentially improve data development and support the development of health care services [116,117]. Palliative care is a growing discipline in the Asia Pacific region, and current research describing digital priorities is limited, although it is acknowledged that digital health can play an important role in supporting education and training [118].

### Limitations

It is possible that recent developments were not reflected in the priority list owing to the ongoing advancement of health care technologies. For example, the FTI trends list is now in its 2021 version and includes new trends, such as home medical laboratory tests and remote metabolic monitoring. Therefore, it is possible that relevant areas were absent from this analysis. Moreover, a weakness of digital health research is the rapid change associated with technology, which may cause the findings of this study to lose relevance over time.

Our decision to reduce the number of trends from 95 to 32 items has broadened the focus of the list, which means that it is possible that more specific and technical areas were not explored in greater depths (eg, faceprints, voiceprints, and chatbots). It is also possible that our Delphi participants will have different views on the priority of some areas post COVID-19, owing to the observed increase in digital health in practice. It is possible that because of the novel nature of some areas, participants gave more priority to familiar areas and therefore, less priority to unfamiliar areas. Questionnaires were mostly completed by participants from English-speaking countries, meaning that the experience of non-English–speaking populations may not be reflected. Specifically, our outcomes may not represent the Asia Pacific and Australasian or African regions, as we had no responses from these areas. Furthermore, the final priority list may not represent non-UK health care systems, as the consensus meeting was only attended by UK residents. We acknowledge that people from different professional backgrounds (including cultures and settings) may assign different levels of priority to trends because of their experiences, work requirements, and personal beliefs. As most participants were clinically focused, it is possible that the priorities were oriented to clinical utility rather than methodology.

### Relevance to Research, Practice, and Policy

Decision makers should ensure that technology is relevant to the needs of palliative care users, as these requirements will influence the design, use, and function of systems [119,120]. For example, health care professionals may generally use technology to access patient data and communicate with other professionals, whereas patients may wish to access their own health data and contact health care services. Further research is needed to develop specific use cases for these scenarios to ensure that the technology can be used meaningfully to achieve the intended outcomes. Furthermore, as the user requirements of people with palliative care needs may differ from those of the general population [121] and because we currently lack resources for widespread implementation of all technologies, it is important that digital health studies provide the data needed to determine best practices and to help identify the barriers and facilitators for adoption.

Researchers should use appropriate methodologies to explore these questions and study associated areas, such as ethical issues, data security, and design. It is important that researchers work with the public, as the comments of the lay representatives in our study (from both the consensus meeting and public engagement workshop) described concerns about the use of personal data. Policy makers should consider issues related to the governance and ethics of current and future digital systems. From a design perspective, we suggest that palliative care professionals collaborate with creative industries (eg, designers, developers, and engineers) to ensure that the designed technologies fulfill the user requirements for specific palliative care use cases.

### Conclusions

The priorities identified in this study represent a wide range of important emerging areas in the fields of digital health, personalized medicine, and data science. Human-centered design and robust governance systems should be considered in future research. Transdisciplinary studies using appropriate methodologies are required to further investigate this priority list. It is important that the risks of using these technologies in palliative care are properly addressed to ensure that these tools are used meaningfully, wisely, and safely and do not cause unintentional harm.

## Appendix

Multimedia Appendix 1 Future Today Institute 2018 Trends List.

Multimedia Appendix 2 Delphi questionnaire (Google forms).

Multimedia Appendix 3 Technology in Palliative Care study scoping review.

Multimedia Appendix 4 Summary of the networks used to invite palliative care professionals to participate.

Multimedia Appendix 5 IQR to be used to guide the level of agreement for Delphi responses.

Multimedia Appendix 6 Level of agreement for each *priority area* following both Delphi rounds.

Multimedia Appendix 7 Voting outcomes for consensus meeting.

Multimedia Appendix 8 Final list of priorities.

Multimedia Appendix 9 Technology in Palliative Care Public Engagement Event information.

Multimedia Appendix 10 Examples of technologies used in palliative care during the COVID-19 pandemic.

## Abbreviations

AI: artificial Intelligence
FTI: Future Today Institute
VR: virtual reality

## References

[1] Topol E (2019). Preparing the healthcare workforce to deliver the digital future the Topol review: an independent report on behalf of the Secretary of State for Health and Social Care. NHS Health Education.

[2] (2018). The future of healthcare: our vision for digital, data and technology in health and care. Department of Health and Social Care.

[3] Haghi M, Thurow K, Stoll R (2017). Wearable devices in medical internet of things: scientific research and commercially available devices. Healthc Inform Res.

[4] Bone AE, Gomes B, Etkind SN, Verne J, Murtagh FE, Evans CJ, Higginson IJ (2018). What is the impact of population ageing on the future provision of end-of-life care? Population-based projections of place of death. Palliat Med.

[5] Barrett M, Combs V, Su JG, Henderson K, Tuffli M, AIR Louisville Collaborative (2018). AIR Louisville: addressing asthma with technology, crowdsourcing, cross-sector collaboration, and policy. Health Aff (Millwood).

[6] Morawski K, Ghazinouri R, Krumme A, Lauffenburger JC, Lu Z, Durfee E, Oley L, Lee J, Mohta N, Haff N, Juusola JL, Choudhry NK (2018). Association of a smartphone application with medication adherence and blood pressure control: the MedISAFE-BP randomized clinical trial. JAMA Intern Med.

[7] Wild SH, Hanley J, Lewis SC, McKnight JA, McCloughan LB, Padfield PL, Parker RA, Paterson M, Pinnock H, Sheikh A, McKinstry B (2016). Supported telemonitoring and glycemic control in people with type 2 diabetes: the Telescot Diabetes Pragmatic Multicenter randomized controlled trial. PLoS Med.

[8] Koehler F, Koehler K, Prescher S, Kirwan B, Wegscheider K, Vettorazzi E, Lezius S, Winkler S, Moeller V, Fiss G, Schleder J, Koehler M, Zugck C, Störk S, Butter C, Prondzinsky R, Spethmann S, Angermann C, Stangl V, Halle M, von Haehling S, Dreger H, Stangl K, Deckwart O, Anker SD (2020). Mortality and morbidity 1 year after stopping a remote patient management intervention: extended follow-up results from the telemedical interventional management in patients with heart failure II (TIM-HF2) randomised trial. Lancet Digit Health.

[9] de Jong MJ, van der Meulen-de Jong AE, Romberg-Camps MJ, Becx MC, Maljaars JP, Cilissen M, van Bodegraven AA, Mahmmod N, Markus T, Hameeteman WM, Dijkstra G, Masclee AA, Boonen A, Winkens B, van Tubergen A, Jonkers DM, Pierik MJ (2017). Telemedicine for management of inflammatory bowel disease (myIBDcoach): a pragmatic, multicentre, randomised controlled trial. Lancet.

[10] Denis F, Yossi S, Septans A, Charron A, Voog E, Dupuis O, Ganem G, Pointreau Y, Letellier C (2017). Improving survival in patients treated for a lung cancer using self-evaluated symptoms reported through a web application. Am J Clin Oncol.

[11] Müller KI, Alstadhaug KB, Bekkelund SI (2017). A randomized trial of telemedicine efficacy and safety for nonacute headaches. Neurology.

[12] Rono HK, Bastawrous A, Macleod D, Wanjala E, Di Tanna GL, Weiss HA, Burton MJ (2018). Smartphone-based screening for visual impairment in Kenyan school children: a cluster randomised controlled trial. Lancet Global Health.

[13] Freeman D, Sheaves B, Goodwin GM, Yu L, Nickless A, Harrison PJ, Emsley R, Luik AI, Foster RG, Wadekar V, Hinds C, Gumley A, Jones R, Lightman S, Jones S, Bentall R, Kinderman P, Rowse G, Brugha T, Blagrove M, Gregory AM, Fleming L, Walklet E, Glazebrook C, Davies EB, Hollis C, Haddock G, John B, Coulson M, Fowler D, Pugh K, Cape J, Moseley P, Brown G, Hughes C, Obonsawin M, Coker S, Watkins E, Schwannauer M, MacMahon K, Siriwardena AN, Espie CA (2017). The effects of improving sleep on mental health (OASIS): a randomised controlled trial with mediation analysis. Lancet Psychiatry.

[14] Kollins SH, DeLoss DJ, Cañadas E, Lutz J, Findling RL, Keefe RS, Epstein JN, Cutler AJ, Faraone SV (2020). A novel digital intervention for actively reducing severity of paediatric ADHD (STARS-ADHD): a randomised controlled trial. Lancet Digital Health.

[15] Craig TK, Rus-Calafell M, Ward T, Leff JP, Huckvale M, Howarth E, Emsley R, Garety PA (2018). AVATAR therapy for auditory verbal hallucinations in people with psychosis: a single-blind, randomised controlled trial. Lancet Psychiatry.

[16] Mariano B (2020). Towards a global strategy on digital health. Bull World Health Organ.

[17] (2020). Global strategy on digital health 2020-2025. World Health Organization.

[18] Nwosu AC, Collins B, Mason S (2017). Big Data analysis to improve care for people living with serious illness: the potential to use new emerging technology in palliative care. Palliat Med.

[19] Hancock S, Preston N, Jones H, Gadoud A (2019). Telehealth in palliative care is being described but not evaluated: a systematic review. BMC Palliat Care.

[20] Nwosu AC, Sturgeon B, McGlinchey T, Goodwin CD, Behera A, Mason S, Stanley S, Payne TR (2019). Robotic technology for palliative and supportive care: strengths, weaknesses, opportunities and threats. Palliat Med.

[21] Archibald MM, Barnard A (2018). Futurism in nursing: technology, robotics and the fundamentals of care. J Clin Nurs.

[22] Jünger S, Payne SA, Brine J, Radbruch L, Brearley SG (2017). Guidance on Conducting and REporting DElphi Studies (CREDES) in palliative care: recommendations based on a methodological systematic review. Palliat Med.

[23] Rowe G, Wright G (2001). Expert opinions in forecasting: the role of the Delphi technique. Principles of Forecasting: A Handbook for Researchers and Practitioners.

[24] Ono R, Wedemeyer DJ (1994). Assessing the validity of the Delphi technique. Futures.

[25] Heiko AV (2008). The Delphi technique for futures research. The Future of Logistics: Scenarios for 2025.

[26] Dalkey N, Brown B, Cochran S (1969). The Delphi Method: An Experimental Study of Group Opinion.

[27] Avella JR (2016). Delphi panels: research design, procedures, advantages, and challenges. Int J Doctor Stud.

[28] Hsu C, Sandford B (2007). The Delphi technique: making sense of consensus. Practical Assess Res Eval.

[29] The Future Today Institute.

[30] (2018). Our data-driven future in healthcare. The Academy of Medical Sciences.

[31] Google Forms.

[32] Jünger S, Payne S, Brearley S, Ploenes V, Radbruch L (2012). Consensus building in palliative care: a Europe-wide Delphi study on common understandings and conceptual differences. J Pain Symptom Manag.

[33] Wherton J, Shaw S, Papoutsi C, Seuren L, Greenhalgh T (2020). Guidance on the introduction and use of video consultations during COVID-19: important lessons from qualitative research. BMJ Leader.

[34] Etkind SN, Bone AE, Lovell N, Cripps RL, Harding R, Higginson IJ, Sleeman KE (2020). The role and response of palliative care and hospice services in epidemics and pandemics: a rapid review to inform practice during the COVID-19 pandemic. J Pain Symptom Manag.

[35] Finucane A, O'Donnell H, Lugton J, Gibson-Watt T, Swenson C, Pagliari C (2021). Digital health interventions in palliative care: a systematic meta-review. NPJ Digit Med.

[36] Chen M, Decary M (2019). Artificial intelligence in healthcare: an essential guide for health leaders. Healthc Manage Forum.

[37] Einav L, Finkelstein A, Mullainathan S, Obermeyer Z (2018). Predictive modeling of U.S. health care spending in late life. Science.

[38] Makar M, Ghassemi M, Cutler DM, Obermeyer Z (2015). Short-term mortality prediction for elderly patients using medicare claims data. Int J Mach Learn Comput.

[39] Sahni N, Simon G, Arora R (2018). Development and validation of machine learning models for prediction of 1-year mortality utilizing electronic medical record data available at the end of hospitalization in multicondition patients: a proof-of-concept study. J Gen Intern Med.

[40] Avati A, Jung K, Harman S, Downing L, Ng A, Shah NH (2018). Improving palliative care with deep learning. BMC Med Inform Decis Mak.

[41] Heintzelman NH, Taylor RJ, Simonsen L, Lustig R, Anderko D, Haythornthwaite JA, Childs LC, Bova GS (2013). Longitudinal analysis of pain in patients with metastatic prostate cancer using natural language processing of medical record text. J Am Med Inform Assoc.

[42] Lodhi M, Stifter J, Yao Y, Ansari R, Kee-Nan G, Wilkie D (2015). Predictive modeling for end-of-life pain outcome using electronic health records. Adv Data Min.

[43] Lodhi M, Ansari R, Yao Y, Keenan G, Wilkie D, Khokhar A (2015). Predictive modeling for comfortable death outcome using electronic health records. Proceedings of the IEEE International Congress on Big Data.

[44] Lindvall C, Lilley EJ, Zupanc SN, Chien I, Udelsman BV, Walling A, Cooper Z, Tulsky JA (2019). Natural language processing to assess end-of-life quality indicators in cancer patients receiving palliative surgery. J Palliat Med.

[45] Chan A, Chien I, Moseley E, Salman S, Bourland SK, Lamas D, Walling AM, Tulsky JA, Lindvall C (2018). Deep learning algorithms to identify documentation of serious illness conversations during intensive care unit admissions. Palliat Med.

[46] Tanuseputro P (2017). Delivering care to those in need: improving palliative care using linked data. Palliat Med.

[47] Selman LE, Chamberlain C, Sowden R, Chao D, Selman D, Taubert M, Braude P (2021). Sadness, despair and anger when a patient dies alone from COVID-19: a thematic content analysis of Twitter data from bereaved family members and friends. Palliat Med.

[48] Nwosu AC, Debattista M, Rooney C, Mason S (2015). Social media and palliative medicine: a retrospective 2-year analysis of global Twitter data to evaluate the use of technology to communicate about issues at the end of life. BMJ Support Palliat Care.

[49] Oliver DP, Washington K, Gage LA, Demiris G (2014). The promise of secret Facebook groups for active family caregivers of hospice patients. J Palliat Med.

[50] Selman LE, Sowden R, Borgstrom E (2021). ‘Saying goodbye’ during the COVID-19 pandemic: a document analysis of online newspapers with implications for end of life care. Palliat Med.

[51] Rajaram A, Morey T, Dosani N, Pou-Prom C, Mamdani M (2019). Palliative care in the twenty-first century: using advanced analytics to uncloak insights from Big Data. J Palliat Med.

[52] Harris J, Cheevers K, Armes J (2018). The emerging role of digital health in monitoring and supporting people living with cancer and the consequences of its treatments. Curr Opin Support Palliat Care.

[53] Nwosu AC, Mason S (2012). Palliative medicine and smartphones: an opportunity for innovation?. BMJ Support Palliat Care.

[54] Bienfait F, Petit M, Pardenaud R, Guineberteau C, Pignon A (2020). Applying m-Health to palliative care: a systematic review on the use of m-Health in monitoring patients with chronic diseases and its transposition in palliative care. Am J Hosp Palliat Care.

[55] Weekly T, Walker N, Beck J, Akers S, Weaver M (2018). A review of apps for calming, relaxation, and mindfulness interventions for pediatric palliative care patients. Children.

[56] Meghani SH, MacKenzie MA, Morgan B, Kang Y, Wasim A, Sayani S (2017). Clinician-targeted mobile apps in palliative care: a systematic review. J Palliat Med.

[57] Phongtankuel V, Adelman RD, Reid MC (2018). Mobile health technology and home hospice care: promise and pitfalls. Progress Palliat Care.

[58] Maggi N, Magnoni LD, Ruggiero C, Gazzarata R, Giacomini M (2019). Information technology system including patient generated health data for cancer clinical care and research. Stud Health Technol Inform.

[59] Sayeed R, Gottlieb D, Mandl KD (2020). SMART Markers: collecting patient-generated health data as a standardized property of health information technology. NPJ Digit Med.

[60] Dudgeon D (2018). The impact of measuring patient-reported outcome measures on quality of and access to palliative care. J Palliat Med.

[61] Johnston B, Flemming K, Narayanasamy MJ, Coole C, Hardy B (2017). Patient reported outcome measures for measuring dignity in palliative and end of life care: a scoping review. BMC Health Serv Res.

[62] Kavalieratos D, Corbelli J, Zhang D, Dionne-Odom JN, Ernecoff NC, Hanmer J, Hoydich ZP, Ikejiani DZ, Klein-Fedyshin M, Zimmermann C, Morton SC, Arnold RM, Heller L, Schenker Y (2016). Association between palliative care and patient and caregiver outcomes. J Am Med Assoc.

[63] Benze G, Nauck F, Alt-Epping B, Gianni G, Bauknecht T, Ettl J, Munte A, Kretzschmar L, Gaertner J (2019). PROutine: a feasibility study assessing surveillance of electronic patient reported outcomes and adherence via smartphone app in advanced cancer. Ann Palliat Med.

[64] Abernethy A, Ahmad A, Zafar S, Wheeler J, Reese J, Lyerly H (2010). Electronic patient-reported data capture as a foundation of rapid learning cancer care. Medical care.

[65] Mandel J, Kreda D, Mandl K, Kohane I, Ramoni R (2016). SMART on FHIR: a standards-based, interoperable apps platform for electronic health records. J Am Med Inform Assoc.

[66] Pavic M, Klaas V, Theile G, Kraft J, Tröster G, Guckenberger M (2020). Feasibility and usability aspects of continuous remote monitoring of health status in palliative cancer patients using wearables. Oncology.

[67] Nwosu AC, Quinn C, Samuels J, Mason S, Payne TR (2018). Wearable smartwatch technology to monitor symptoms in advanced illness. BMJ Support Palliat Care.

[68] Chirico A, Lucidi F, De Laurentiis M, Milanese C, Napoli A, Giordano A (2016). Virtual reality in health system: beyond entertainment. A mini-review on the efficacy of VR during cancer treatment. J Cell Physiol.

[69] Johnson T, Bauler L, Vos D, Hifko A, Garg P, Ahmed M, Raphelson M (2020). Virtual reality use for symptom management in palliative care: a pilot study to assess user perceptions. J Palliat Med.

[70] Niki K, Okamoto Y, Maeda I, Mori I, Ishii R, Matsuda Y, Takagi T, Uejima E (2019). A novel palliative care approach using virtual reality for improving various symptoms of terminal cancer patients: a preliminary prospective, multicenter study. J Palliat Med.

[71] Austin P, Lovell M, Siddall P (2019). The efficacy of virtual reality for persistent cancer pain: a call for research. J Pain Symptom Manag.

[72] Hsieh W (2020). Virtual reality video promotes effectiveness in advance care planning. BMC Palliat Care.

[73] Evans L, Taubert M (2018). State of the science: the doll is dead: simulation in palliative care education. BMJ Support Palliat Care.

[74] Lee AL, DeBest M, Koeniger-Donohue R, Strowman SR, Mitchell SE (2019). The feasibility and acceptability of using virtual world technology for interprofessional education in palliative care: a mixed methods study. J Interprofess Care.

[75] Clabburn O, Groves KE, Jack B (2020). Virtual learning environment (‘Ivy Street’) for palliative medicine education: student and facilitator evaluation. BMJ Support Palliat Care.

[76] Liu L, Stroulia E, Nikolaidis I, Miguel-Cruz A, Rios Rincon A (2016). Smart homes and home health monitoring technologies for older adults: a systematic review. Int J Med Inform.

[77] Pavic M, Klaas V, Theile G, Kraft J, Tröster G, Blum D, Guckenberger M (2020). Mobile health technologies for continuous monitoring of cancer patients in palliative care aiming to predict health status deterioration: a feasibility study. J Palliat Med.

[78] Pantelopoulos A, Bourbakis N (2010). A survey on wearable sensor-based systems for health monitoring and prognosis. IEEE Trans Syst Man Cybern.

[79] Di Sanzo M, Cipolloni L, Borro M, La Russa R, Santurro A, Scopetti M, Simmaco M, Frati P (2017). Clinical applications of personalized medicine: a new paradigm and challenge. Curr Pharm Biotechnol.

[80] Reid VL, McDonald R, Nwosu AC, Mason SR, Probert C, Ellershaw JE, Coyle S (2017). A systematically structured review of biomarkers of dying in cancer patients in the last months of life; an exploration of the biology of dying. PLoS ONE.

[81] Coyle S, Scott A, Nwosu AC, Latten R, Wilson J, Mayland CR, Mason S, Probert C, Ellershaw J (2016). Collecting biological material from palliative care patients in the last weeks of life: a feasibility study. BMJ Open.

[82] Kim J, Lee Y, Kwak M, Jun G, Koh E, Song S, Seong J, Kim JW, Kim K, Kim S, Hwang D (2014). Metabolomics approach to serum biomarker for loperamide-induced constipation in SD rats. Lab Anim Res.

[83] Amgarth‐Duff I, Hosie A, Caplan G, Agar M (2020). Toward best practice methods for delirium biomarker studies: an international modified Delphi study. Int J Geriatr Psychiatry.

[84] DeMarshall C, Oh E, Kheirkhah R, Sieber F, Zetterberg H, Blennow K, Nagele RG (2019). Detection of early-stage Alzheimer’s pathology using blood-based autoantibody biomarkers in elderly hip fracture repair patients. PLoS ONE.

[85] Fladvad T, Klepstad P, Langaas M, Dale O, Kaasa S, Caraceni A (2013). Variability in UDP-glucuronosyltransferase genes and morphine metabolism: observations from a cross-sectional multicenter study in advanced cancer patients with pain. Pharmacogenet Genom.

[86] Barratt D, Bandak B, Klepstad P, Dale O, Kaasa S, Christrup L (2014). Genetic, pathological and physiological determinants of transdermal fentanyl pharmacokinetics in 620 cancer patients of the EPOS study. Pharmacogenet Genom.

[87] Klepstad P, Fladvad T, Skorpen F, Bjordal K, Caraceni A, Dale O (2011). Influence from genetic variability on opioid use for cancer pain: a European genetic association study of 2294 cancer pain patients. Pain.

[88] DeSanto-Madeya S, Tjia J, Fitch C, Wachholtz A (2020). Feasibility and acceptability of digital legacy-making: an innovative story-telling intervention for adults with cancer. Am J Hosp Palliat Care.

[89] Taubert M, Watts G, Boland J, Radbruch L (2014). Palliative social media. BMJ Support Palliat Care.

[90] Clabburn O, Knighting K, Jack BA, O’Brien MR (2019). The use of digital legacies with people affected by motor neurone disease for continuing bonds: an interpretative phenomenological analysis study. Palliat Med.

[91] Taubert M, Norris J, Edwards S, Snow V, Finlay IG (2018). Talk CPR - a technology project to improve communication in do not attempt cardiopulmonary resuscitation decisions in palliative illness. BMC Palliat Care.

[92] Coop H, Marlow C (2019). Do we prepare patients for their digital legacy? A survey of palliative care professionals. Palliat Med.

[93] Calton B, Abedini N, Fratkin M (2020). Telemedicine in the time of coronavirus. J Pain Symptom Manag.

[94] Grewal US, Terauchi S, Beg MS (2020). Telehealth and palliative care for patients with cancer: implications of the COVID-19 pandemic. JMIR Cancer.

[95] Ritchey KC, Foy A, McArdel E, Gruenewald DA (2020). Reinventing palliative care delivery in the era of COVID-19: how telemedicine can support end of life care. Am J Hosp Palliat Care.

[96] Bettini EA (2020). COVID-19 pandemic restrictions and the use of technology for pediatric palliative care in the acute care setting. J Hosp Palliat Nurs.

[97] Chávarri-Guerra Y, Ramos-López WA, Covarrubias-Gómez A, Sánchez-Román S, Quiroz-Friedman P, Alcocer-Castillejos N, Milke-García M, Carrillo-Soto M, Morales-Alfaro A, Medina-Palma M, Aguilar-Velazco J, Morales-Barba K, Razcon-Echegaray A, Maldonado J, Soto-Perez-de-Celis E (2021). Providing supportive and palliative care using telemedicine for patients with advanced cancer during the COVID-19 pandemic in Mexico. Oncologist.

[98] Mackey RM, Yeow ME, Christensen AR, Ingram C, Carey EC, Lapid MI (2020). Reconnecting: strategies for supporting isolated older adults during COVID-19 through tele-palliative care. Clin Gerontol.

[99] Harris DA, Archbald-Pannone L, Kaur J, Cattell-Gordon D, Rheuban KS, Ombres RL, Albero K, Steele R, Bell TD, Mutter JB (2021). Rapid telehealth-centered response to COVID-19 outbreaks in postacute and long-term care facilities. Telemed e-Health.

[100] Flores S, Abrukin L, Jiang L, Titone L, Firew T, Lee J, Gavin N, Romney M, Nakagawa S, Chang BP (2020). Novel use of telepalliative care in a New York City emergency department during the COVID-19 pandemic. The J Emerg Med.

[101] Lu Y, Xie D, Zhang X, Dong S, Zhang H, Yu B, Wang G, Wang JJ, Li L (2020). Management of intractable pain in patients with implanted spinal cord stimulation devices during the COVID-19 pandemic using a remote and wireless programming system. Front Neurosci.

[102] Sansom‐Daly UM, Bradford N (2020). Grappling with the “human” problem hiding behind the technology: telehealth during and beyond COVID‐19. Psycho‐Oncol.

[103] Samara J, Liu W, Kroon W, Harvie B, Hingeley R, Johnston N (2021). Telehealth palliative care needs rounds during a pandemic. J Nurse Practition.

[104] Chua IS, Jackson V, Kamdar M (2020). Webside manner during the COVID-19 pandemic: maintaining human connection during virtual visits. J Palliat Med.

[105] Lally K, Kematick BS, Gorman D, Tulsky J (2021). Rapid conversion of a palliative care outpatient clinic to telehealth. JCO Oncol Pract.

[106] Crosby B, Hanchanale S, Stanley S, Nwosu AC (2021). Evaluating the use of video communication technology in a hospital specialist palliative care team during the COVID-19 pandemic. AMRC Open Res.

[107] Lal A, Bell G, Curseen K, Kavalieratos D (2021). Teaching telepalliative care: an elective rotation for medical students during the COVID-19 pandemic. J Palliat Med.

[108] Abel J, Taubert M (2020). Coronavirus pandemic: compassionate communities and information technology. BMJ Support Palliat Care.

[109] Mills J, Li C, Fullerton S, Chapman M, Jap J, Sinclair C, Collins A, Campbell E (2020). Staying connected and informed: online resources and virtual communities of practice supporting palliative care during the novel coronavirus pandemic. Progress Palliat Care.

[110] Niki K, Okamoto Y, Ueda M (2020). Response to Wang et al., virtual reality as a bridge in palliative care during COVID-19 (DOI: 10.1089/jpm.2020.0212). J Palliat Med.

[111] Wang SS, Teo WZ, Teo WZ, Chai YW (2020). Virtual reality as a bridge in palliative care during COVID-19. J Palliat Med.

[112] Niki K, Yahara M, Inagaki M, Takahashi N, Watanabe A, Okuda T, Ueda M, Iwai D, Sato K, Ito T (2020). Immersive virtual reality reminiscence reduces anxiety in the oldest-old without causing serious side effects: a single-center, pilot, and randomized crossover study. Front Hum Neurosci.

[113] Posner G, Maniate J, Dale-Tam J, Endres K, Corral J (2020). Virtual reality videos for training and protocol dissemination during a pandemic. MedEdPublish.

[114] Mills J, Fox J, Damarell R, Tieman J, Yates P (2021). Palliative care providers' use of digital health and perspectives on technological innovation: a national study. BMC Palliat Care.

[115] Allsop MJ, Powell RA, Namisango E (2018). The state of mHealth development and use by palliative care services in sub-Saharan Africa: a systematic review of the literature. BMJ Support Palliat Care.

[116] Nkhoma KB, Ebenso B, Akeju D, Adejoh S, Bennett M, Chirenje M, Dandadzi A, Nabirye E, Namukwaya E, Namisango E, Okunade K, Salako O, Harding R, Allsop MJ (2021). Stakeholder perspectives and requirements to guide the development of digital technology for palliative cancer services: a multi-country, cross-sectional, qualitative study in Nigeria, Uganda and Zimbabwe. BMC Palliat Care.

[117] Ngoma M, Mushi B, Morse RS, Ngoma T, Mahuna H, Lambden K, Quinn E, Sagan SB, Ho YX, Lucas FL, Mmari J, Miesfeldt S (2021). Mpalliative care link: examination of a mobile solution to palliative care coordination among Tanzanian patients with cancer. JCO Global Oncol.

[118] Mills J, Kim S, Chan H, Ho M, Montayre J, Liu M, Lin C (2021). Palliative care education in the Asia Pacific: challenges and progress towards palliative care development. Progress Palliat Care.

[119] Dolan H, Eggett C, Holliday L, Delves S, Parkes D, Sutherland K (2021). Virtual care in end of life and palliative care: a rapid evidence check. J Telemed Telecare.

[120] Disalvo D, Agar M, Caplan G, Murtagh FE, Luckett T, Heneka N, Hickman L, Kinchin I, Trethewie S, Sheehan C, Urban K, Cohen J, Harlum J, Long B, Parker T, Schaefer I, Phillips J (2021). Virtual models of care for people with palliative care needs living in their own home: a systematic meta-review and narrative synthesis. Palliat Med.

[121] Etkind SN, Bone AE, Gomes B, Lovell N, Evans CJ, Higginson IJ, Murtagh FE (2017). How many people will need palliative care in 2040? Past trends, future projections and implications for services. BMC Med.

[122] Marie Curie.

