# Supplementary material for: Identification of Digital Health Priorities for Palliative Care Research: Modified Delphi Study
Source: JMIR Aging. 2022 Mar 21;5(1):e32075. doi: 10.2196/32075 (PMC9090235; doi:10.2196/32075)
Supplement: Multimedia Appendix 1 [file aging_v5i1e32075_app1.pdf]

TABLE OF CONTENTS

02 Welcome Letter

08 Executive Summary

08 Key Takeaways

10 Guide To The Year Ahead

12 Methodology

15 How To Use Our Report

16 Ten Important Questions

22 The Most Important Tech Trends For Your Industry And Organization

47 Artificial Intelligence

51 The AI Cloud

51 Proprietary, Homegrown AI Languages

51 AI Chipsets

52 Cognitive Computing

52 Bots

52 Marketplaces For AI Algorithms

52 More Consolidation in AI

53 Consumer-Grade AI Applications Debut

53 Digital Assistants Become Ubiquitous

53 A Bigger Role For Ambient Interfaces

53 Deep Linking Everywhere

54 Making AI Explain Itself

54 Accountability and Trust

54 Hidden Bias Leads To Big Problems

55 China's AI Boom

56 Real-Time Machine Learning

56 Natural Language Understanding (NLU)

56 Machine Reading Comprehension (MRC)

56 Natural Language Generation (NLG)

56 Generative Algorithms For Voice, Sound and Video

57 Image Completion

57 Predictive Machine Vision

57 Much Faster Deep Learning

57 Reinforcement Learning and Hierarchical RL

57 Continuous Learning

58 Multitask Learning

58 Adversarial Machine Learning

59 Recognition Technologies

60 Faceprints

61 Voiceprints

62 Wifi Tracking

63 Gesture Recognition

64 Personality and Character Recognition

65 Ambient Proximity

66 Hidden Bias in Recognition Technologies

67 Security, Privacy and Data

69 Compliance Challenges and Unrealistic Budgets

69 DDoS Attacks Will Increase

69 Ransomware As A Service

70 Russia's Gifted Hacker Community Grows

70 New Infrastructure Targets

70 Hacktivism On The Rise

71 Third-Party Verified Identities

71 Targeted Attacks on Digital Assistants

71 Zero-Knowledge Proofs Go Commercial

71 Zero-Day Exploits On The Rise

71 Backdoors

73 Remote Kill Switches

73 AI-Powered Automated Hacking

73 Offensive Government Hacking

73 More Cyber Mission Forces in the Field

73 Strange Computer Glitches Will Keep Happening

74 Proliferation of Darknets, Aided By Cryptocurrencies

74 New Open Source App Vulnerabilities

74 Selfie Security Using Faceprints

74 Bounty Programs

79 The General Data Protection Regulation Takes Effect

79 Right To Eavesdrop/ Be Eavesdropped On

79 Defining What Constitutes Online Harassment

80 Drone Surveillance

80 Personal and One-To-Few Networks

80 Leaking

80 Blocking the Ad Blockers

81 Organizational Doxing

81 Anonymity

81 Authenticity

81 Differential Privacy

82 Anti-Recognition Cammo and Glasses

82 Digital Self-Incrimination

82 SWATting at Trolls

83 Revenge Porn

83 Eye In The Sky

83 Law Enforcement Using Recognition Algorithms To ID Faces

84 Data Retention Policies

84 Encryption Management

© 2018 FUTURE TODAY INSTITUTE

84 Who Owns Your Personal, Biometric Data?
85 Global Data Scientist Shortages

86 Advanced Robotics
87 Self-Assembling Robots
88 Robot Compilers
89 Molecular Robotics
90 Collaborative Robotics
91 Ethical Manufacturing
92 Soft Robotics
93 Human-Machine Interfaces
94 Smart Dust
95 Personal Robots and Butlers
97 Robot Abuse
98 3D Printing

99 Transportation
100 Flying Taxis
100 Autonomous Underwater Vehicles (AUVs)
100 Drone Delivery
101 Increasing Patents and Calls For New Regulation
102 Drone Lanes
102 Personal Home Drone Surveillance
102 Sense And Avoid Technology
102 Microdrones and Drones Used In Dangerous/Hard-To-Reach Areas
102 Drone Swarms
102 Clandestine, Disappearing Drones
103 Autonomous Vehicle Testing In Cities Around the U.S.
103 Armchair AV Mechanics
103 Assisted Driving Before Full Automation
104 Adaptive Driving Systems
104 Vehicle-to-Vehicle (V2V) Communications
104 Electric Vehicles
104 Cars as Interfaces
104 Solar Highways
105 Autonomous Vehicle Legislation
105 Flying Cars
106 Flights
107 Autonomous Ships

108 News Media, Book Publishing, Social Networks and the First Amendment
110 Natural Language Generation for Reading Levels
111 Computational Photography
112 Computational Journalism
113 I-Teams For Algorithms and Data
114 Voice Interfaces For News and Books
115 Proximity News
116 Crowdlearning
117 Digital Frailty
119 Radical Transparency
120 Limited-Edition News Products
121 One-To-Few Publishing
122 Notification Layer (A Tragedy of the Commons)
123 Journalism as a Service (JaaS)
124 Transparency in Metrics
125 Real-Time Fact Checking
126 Offline Is The New Online
127 Audio Search Engines
128 Synthetic Data Sets
129 Connected TVs
129 Decentralizing The Web
129 Streaming Social Video
130 New Video and Audio Story Formats
131 Media Consolidation
132 Tweaks To Social Network Algorithms
133 The First Amendment in a Digital Age

134 Entertainment Media
135 Holograms
135 Virtual Reality
136 360-degree Video
136 Augmented Reality
137 Mixed Reality Arcades
138 MMOMRGs

141 Marketing and Advertising Technologies
142 VR For Marketing
143 AI For the Creative Process
144 FOBO
145 Retail APIs
146 Digital Associates

147 Energy
148 Green Tech
149 Charging Stations
150 Ultra-High-Voltage Direct Current and Macro Grids
151 Better Batteries

152 Climate And Geoscience
153 Anthropocene
155 Extreme Weather Events
157 Human Migration Patterns Shift
158 Geoengineering

159 Agricultural Technologies
160 Indoor and Outdoor Plant Factories and Microfarms
161 Deep Learning For Farming and Food Recognition
162 Smart Farms
163 Terraforming
164 Cultivated Food and Beverage

165 Biotechnologies, Genomic Editing and Biointerfaces
170 Genome Editing
170 Biological DVRs
170 Human DNA-Powered Devices
170 Using Our DNA As Hard Drives
170 Nanobot Nurses
170 Custom-Crafted Microbes

© 2018 FUTURE TODAY INSTITUTE

171 Precision Medicine Just For You
171 Running Out Of Space For Genome Storage
171 Genome Editing Research Clashes With Public Opinion
171 Nootropics and Neuroenhancers
172 Microbiome Extinction
173 Building A Comprehensive Human Cell Atlas
174 Biointerfaces Laminated Onto Our Skin

175 Health Technologies and Wearables
177 Patient-Generated Health Data
178 Touch-Sensitive Prosthetics
179 Smart Thread
180 Bioelectronics
181 Wearables
181 Smart Glasses
181 Hearables / Earables
181 Head Mounted Displays
181 Smart Bras
182 Smart Helmets
182 Smart Gloves
182 Tattooables
183 Thinkables
183 Ingestables
183 Smart Fabrics
183 Smartwatches
183 Smart Shoes
183 Wireless Body Area Networks

184 Smart Homes and the Internet Of Things
186 Privacy Laws, Net Neutrality and Hackers Threaten the Internet of Things
188 Searching The Internet of Physical Things
189 Intelligent Cameras
190 Our Appliances Will Have Digital Assistants
190 Smart Appliance Screens Are Coming
190 Home Appliances Will Talk To Each Other
190 Wireless Kitchens
191 Smarter Home Security
191 Smart Remotes
191 Smart Mirrors
191 Our Smarthomes Become Weaponized

192 Workplace and Learning Technologies
193 Universal Basic Income (UBI)
194 AI in Hiring
196 Productivity Bots
198 Adaptive Learning
199 Nanodegrees
200 Sharing Economy & Lendership

201 Financial Technologies and Cryptocurrencies
202 E-Resident and Location-Independent Digital Business Identity Cards
204 Social Payments
205 Cryptocurrencies
207 Blockchain
208 Open Banking
209 Financial Inclusion and Targeting the Underbanked

210 Smart Cities
213 Smart City Initiatives
214 Faster Connectivity With 5G
215 City-Level Cyber Security

217 Government And Technology Policy
218 Splinternets
219 Election Security
220 Anti-Trust Lawsuits
221 Old Laws Clash With New Technology
223 Digital Caliphate
224 Governments Asking Tech Companies To Help Fight the Spread of Misinformation, Propaganda and Terrorism
225 Overhauling Government Tech Infrastructure

227 Space
228 Commercial Space Flight
229 CubeSats
230 Asteroid Mining For Resources
231 Space Exploration

232 Ten Weak Signals For 2019

235 About the Authors

236 About The Future Today Institute

237 Disclaimer

238 The Signals Are Talking

239 Companies, Organizations, Universities and Government Agencies Mentioned In Our 2018 Trends Report.

247 Contact Information

© 2018 FUTURE TODAY INSTITUTE
