# Supplementary material for: Identification of Digital Health Priorities for Palliative Care Research: Modified Delphi Study
Source: JMIR Aging. 2022 Mar 21;5(1):e32075. doi: 10.2196/32075 (PMC9090235; doi:10.2196/32075)
Supplement: Multimedia Appendix 4 [file aging_v5i1e32075_app4.pdf]

Appendix: Summary of the networks used to invite palliative care professionals to participate

Group(s) contacted	Method	Approximate reach (n)
International Collaborative for Best Care of the Dying Person (a multidisciplinary, international palliative care research group; https://bestcareforthedying.org)	Individuals contacted by email via administrator	79
CHAIN (Contact, Help, Advice and Information Network; https://www.networks.nhs.uk/nhs-networks/chain) UK National Health Service (NHS) technology interest email list.	Individuals contacted by email via administrator	8326
Nine Marie Curie Hospices UK	Individuals contacted by email via local administrators and research leads.	450
NHS England email distribution lists of the following: Allied Health Professionals (National); Healthcare Science Practitioners	Individuals contacted by email via administrator	1000

(National); Psychological professions (North); Cancer alliances (North); Genomics network (National); Informatics and bioinformatics (North West).		
Social media	Four tweets on Twitter were sent from ACN’s Twitter profile.	12058
Technology in Palliative Care Special Interest Group (https://amaranwosu.com/pads)	Targeted email to professionals from a palliative care technology event who had previously provided consent to receive information about future studies	57
