# Supplementary material for: Identification of Digital Health Priorities for Palliative Care Research: Modified Delphi Study
Source: JMIR Aging. 2022 Mar 21;5(1):e32075. doi: 10.2196/32075 (PMC9090235; doi:10.2196/32075)
Supplement: Multimedia Appendix 5 [file aging_v5i1e32075_app5.pdf]

Appendix: Interquartile Range to be used to guide the Level of Agreement for Delphi responses

Level of Agreement

- “Very high agreement” – median 5; percentage agreement ≥80%; IQR 0
- “High agreement” – median 4/5; percentage agreement ≥80%; IQR 1
- “Moderate agreement” – median ≤4; percentage agreement 60–79%; IQR 1
- “Low agreement” – median <4; percentage agreement $<60\%$; IQR >1
