# Supplementary material for: Identification of Digital Health Priorities for Palliative Care Research: Modified Delphi Study
Source: JMIR Aging. 2022 Mar 21;5(1):e32075. doi: 10.2196/32075 (PMC9090235; doi:10.2196/32075)
Supplement: Multimedia Appendix 6 [file aging_v5i1e32075_app6.pdf]

Appendix: Level of agreement for each 'priority area' following both Delphi rounds

		Round 1				Round 2			
**Q**	**Description**	**Median Rating (n=66)**	**IQR**	**% Agreement (4/5 on scale)**	**Level of Agreement**	**Median Rating (n=50)**	**IQR**	**% Agreement (4/5 on scale)**	**Level of Agreement**
**1.1**	Development of Electronic Health Records (EHR) and systems based approaches to collection and utilisation of Big Data	4	1	76%	Moderate	1	4	80%	High
**1.2**	Patient-Generated Health Data (PGHD) – what to collect and how: harnessing and using 'physical function' data via sensor-based technologies (edited)	4	1	60%	Moderate	1	4	62%	Moderate
**1.3**	Governance in the use of 'Big Data' – regulation and responsibility	5	1	83%	High	1	5	80%	High
**1.4**	Cybersecurity and ransomware – Packaging, capturing and transferring data and keeping it safe	5	1	80%	High	1	5	82%	High
**1.5**	Ethical Challenges in Big Data Health Research: implications for informed consent and participation	4	1	78%	Moderate	1	5	82%	High
**2.1**	Smart Threads – early development experimental stages but with positive results as a potential diagnostic or monitoring technology, or as a mechanism for displaying information	3	1	45%	Low	1	3	42%	Low
**2.2**	Physical Activity Change Detection (PACD)	3	1	45%	Low	1	3	36%	Low
**2.3**	Reducing burdensome interventions for patients: optimising monitoring and recording of physiological signals	4	1	82%	High	2	4	75%	Low

**2.4**	Wearable Health Trackers	3	1	44%	Low	1	3	36%	Low
**2.5**	Wearable fabrics for the detection of stimuli (physical functioning e.g. temperature, motion, strain, activity monitoring)	3	2	36%	Low	2	3	27%	Low
**2.6**	Body area network (BAN) technology	3	2	30%	Low	2	3	27%	Low
**2.7**	Wearable technology – privacy and ethical considerations	4	2	67%	Low	2	4	69%	Low
**3.1**	Use of mobile devices to gather patient reported symptom outcomes (PRO's)/Patient Performance Status	4	1	81%	High	1	4	89%	High
**3.2**	App design: Clinical input – Safety, efficacy, accuracy and assessment of risk	4	1	76%	Moderate	1	4	85%	High
**3.3**	Portable Hospital-level screening/diagnostics in the home	4	2	73%	Low	2	4	73%	Low
**4.1**	Telehealth/eHealth to support patients and their families in their own homes	5	1	92%	High	1	5	89%	High
**4.2**	Telehealth/eHealth for electronic patient-reported outcomes (ePRO)	5	1	83%	High	1	4	84%	High
**5.1**	Virtual reality (VR): Distraction therapy to ameliorate symptoms – pain, distress, anxiety	4	2	73%	Low	2	4	73%	Low
**5.2**	VR: grief and bereavement following the death of a patient	4	2	50%	Low	1	4	55%	Low
**5.3**	VR: for education and training needs	4	1	80%	High	1	4	82%	High
**6.1**	Artificial intelligence (AI) and Machine Learning (ML) to improve outcomes for individuals: Natural Language Processing (NLP) and systems-based approaches for prediction and 'screening'	4	1	62%	Moderate	1	4	56%	Low
**6.2**	Role of Big Data and AI/ML for Population Health Management – population level data	4	1	62%	Moderate	1	4	58%	Low
**6.3**	AI/ML – automation of human processes: ethical and moral issues	4	2	58%	Low	2	4	69%	Low
**7.1**	Robotics – for assistance and daily living	4	1	50%	Low	1	4	56%	Low

**7.2**	Robotics – for companionship/social inclusion	3	2	40%	Low	2	3	33%	Low
**7.3**	Robotics – optimisation of surgery	4	2	64%	Low	2	4	75%	Low
**7.4**	Robotics – Education	4	1	53%	Low	1	4	58%	Low
**8.1**	Smart Home Sensors – detect changes in health condition/physical function	4	2	64%	Low	1	4	65%	Moderate
**8.2**	Smart Home Sensors – Alert systems and monitoring – home security and controls	4	2	64%	Low	2	4	65%	Low
**8.3**	Smart Cities – Built Environment and Big Data	3	1	41%	Low	1	3	47%	Low
**9.1**	Genome profiling and Personalised Medicine	4	2	66%	Low	2	4	62%	Low
**9.2**	Genetic editing and biomarker technology for earlier disease detection and possible disease prevention	4	2	68%	Low	2	4	58%	Low
