# Supplementary material for: Identification of Digital Health Priorities for Palliative Care Research: Modified Delphi Study
Source: JMIR Aging. 2022 Mar 21;5(1):e32075. doi: 10.2196/32075 (PMC9090235; doi:10.2196/32075)
Supplement: Multimedia Appendix 7 [file aging_v5i1e32075_app7.pdf]

Appendix: Voting outcomes for consensus meeting

High Agreement		Group 1	Group 2	Deciding vote outcome (n=11)
1	Development of Electronic Health Records (EHR) and systems-based approaches to collection and utilisation of Big Data	In	In	NA
2	Governance in the use of 'Big Data' – regulation and responsibility	In	In	Included combined with item 3
3	Cybersecurity and ransomware – Packaging, capturing and transferring data and keeping it safe	In	Out	Included (combined) 11/11<br><br>Combined with item 2
4	Ethical Challenges in Big Data Health Research: implications for informed consent and participation	In	Out	11/11

5	Use of mobile devices to gather patient reported symptom outcomes (PRO's)/Patient Performance Status	In	In	NA
6	App design: Clinical input – Safety, efficacy, accuracy and assessment of risk	In	In	NA
7	Telehealth/eHealth to support patients and their families in their own homes	In	In	Combined with item 8
8	Telehealth/eHealth for electronic patient-reported outcomes (ePRO)	In	Out	Included 11/11<br><br>combined with item 7
9	VR: for education and training needs	Out	Out	
**Moderate Agreement**		**Group 1**	**Group 2**	**Vote / Outcome**
**10**	Patient-Generated Health Data (PGHD) – what to collect and how: harnessing and using 'physical function' data via sensor-based technologies (edited)	Out	In	Included 11/11
**11**	Smart Home Sensors – detect changes in health condition/physical function	Out	In	Included 6/11

**Low Agreement**		**Group 1**	**Group 2**	**Vote / Outcome**
**12**	Smart Threads – early development experimental stages but with positive results as a potential diagnostic or monitoring technology, or as a mechanism for displaying information	Out	Out	NA
**13**	Physical Activity Change Detection (PACD)	Out	In	Included (combined) 10/11 combined with item 15
**14**	Reducing burdensome interventions for patients: optimising monitoring and recording of physiological signals	Out	Out	NA
**15**	Wearable Health Trackers	Out	In	Included (combined) 9/11 combined with item 13
**16**	Wearable fabrics for the detection of stimuli (physical functioning e.g. temperature, motion, strain, activity monitoring)	Out	Out	NA

**17**	Body area network (BAN) technology	Out	Out	NA
**18**	Wearable technology – privacy and ethical considerations	Out	Out	NA
**19**	Portable Hospital-level screening/diagnostics in the home	Out	Out	NA
**20**	VR: Distraction therapy to ameliorate symptoms – pain, distress, anxiety	In	In	NA
**21**	VR: grief and bereavement following the death of a patient	Out	Out	NA
**22**	AI and ML to improve outcomes for individuals: Natural Language Processing (NLP) and systems-based approaches for ‘prediction’ and ‘screening	In	In	NA
**23**	Role of ‘Big Data’ and AI/ML for Population Health Management – population level data	In	In	NA
**24**	AI/ML – automation of human processes: ethical and moral issues	In	In	NA
**25**	Robotics – for assistance and daily living	Out	Out	NA
**26**	Robotics – for companionship/social inclusion	Out	Out	NA
**27**	Robotics – optimisation of surgery	Out	Out	NA

**28**	Robotics – Education (including simulation)	Out	Out	NA
**29**	Smart Home Sensors – Alert systems and monitoring – home security and controls	Out	Out	NA
**30**	Smart Cities – Built Environment and Big Data	Out	Out	NA
**31**	Genome profiling and Personalised Medicine	In	In	NA
**32**	Genetic editing and biomarker technology for earlier disease detection and possible disease prevention	In<br><br>Focus on biomarkers rather than genetic editing.	Out	Included 11/11
