# Supplementary material for: Identification of Digital Health Priorities for Palliative Care Research: Modified Delphi Study
Source: JMIR Aging. 2022 Mar 21;5(1):e32075. doi: 10.2196/32075 (PMC9090235; doi:10.2196/32075)
Supplement: Multimedia Appendix 8 [file aging_v5i1e32075_app8.pdf]

Appendix. Final list of priorities.

Topic area	Priority	Potential areas for further study identified by public engagement group
Telehealth and telemedicine	Use of telehealth and telemedicine to support patients and caregivers	• How can the telehealth systems be best used to provide remote support for patients and caregivers?<br>• How can video-calling technology be used by health professionals to deliver palliative care?
**AI**[a]		
	The use of different AI methodologies (eg, machine learning, NLP[b], deep learning, and neural networks) for prediction and screening in palliative care	• How can algorithms for prediction and screening be developed safely and effectively for palliative care patients?<br>• How can algorithmic-driven data be used for palliative care research?<br>• What are the ethical and legal issues concerning use of AI in palliative care?

		• How can bias be prevented, identified, and addressed?
	Ethical and moral issues concerning use of AI in palliative care	• What are the ethical, legal, security and privacy issues of using AI palliative care?<br>• How can bias in AI apps be identified and addressed?<br>• Who is responsible for maintaining trust in using AI in palliative care?
Big data		
	Collection and use of big data, from EHR[c] systems	• How can EHR be best designed to optimize use of big data in palliative care?<br>• How can big data be used to improve palliative care at an individual and population health perspective?<br>• What are the education needs of staff regarding the use of big data in palliative care?

	Governance, data security, and regulation of big data use in palliative care	• What are the responsibilities of stakeholders in the design and use of big data, across different aspects of palliative care?<br>• What data security considerations are required for the use of big data in palliative care?
	Ethical challenges of big data health research	• What are the ethical issues in palliative care research using big data?<br>• What are the implications for informed consent and participation in big data research?
	Role of *big data* and AI for palliative care population health management	• How can novel data analysis methods use population level data to support palliative care?
**Mobile devices and wearables**		
	Use of mobile devices to support communication, patient monitoring, and PROMs[d]	• How can data from mobile devices be used to monitor physical and emotional well-being?

		• How can mobile devices support the collection of PROMs[d]?<br>• How can mobile devices be used to support communication and information sharing with patients, caregivers, and health professionals?<br>• How can mobile devices be used for therapeutic care delivery?<br>• How can advance care planning discussions be best supported, documented, and shared.
	Development of apps for clinical use in palliative care	• How can apps be designed to ensure safety, efficacy, and accuracy?<br>• What are the interoperability considerations of app design/development?<br>• How can risks of app assessment be identified and managed?
	PGHD[e] to promote personalized palliative care	• What data should be collected and what mechanisms should be used for this?

		• How can sensor-based technologies be best used to support generation of PGHD?<br>• How can PGHD be used for active and passive palliative care management?
	Wearable Health Trackers for PACD[f]	• How can wearable health trackers support physical activity for people with palliative care needs?<br>• Can wearable technologies be used to detect physical decline in serious illness?<br>• Can wearable technologies help self-management of palliative illness?
VR[g]	Use of VR for symptom management in palliative care	• How can VR be used for symptom management in palliative care?<br>• What VR equipment, processes, and systems offer the best experience for users?

The Smart Home	Use of Smart Home technologies (eg, internet of things) and sensors for monitoring of health status	• How can the internet of things technologies be used to provide palliative care at home?<br>• How can smart (home) assistants support palliative care delivery?<br>• What are the privacy, ethical, and legal issues related to the smart home in palliative care?
**Biotechnology**		
	Genome profiling and personalized medicine	• How can personalized medicine be used to improve symptom management or disease specific management in palliative care?
	Genetic editing and biomarker technology for earlier disease detection and possible disease management/prevention	• What palliative care complications could potentially benefit from early detection or prevention (eg, to predict individuals susceptible to development of metastases, pathological fracture, or hypercalcemia)?

		• Can genetic editing be used to improve management for palliative care?
Digital legacy	Use of technologies that contribute to digital legacy in palliative care	• How can different forms of digital material be used actively to support patients and caregivers to create a *digital legacy*?<br>• How should digital legacy be managed after death?<br>• What are the potential risks and ethical issues related to digital legacy?

[a]AI: artificial intelligence.

[b]NLP: natural language processing.

[c]EHR: electronic health record.

[d]PROM: patient-reported outcome measure.

[e]PGHD: patient-generated health data.

[f]PACD: physical activity change detection.

[g]VR: virtual reality.
