# Supplementary material for: Identification of Digital Health Priorities for Palliative Care Research: Modified Delphi Study
Source: JMIR Aging. 2022 Mar 21;5(1):e32075. doi: 10.2196/32075 (PMC9090235; doi:10.2196/32075)
Supplement: Multimedia Appendix 9 [file aging_v5i1e32075_app9.pdf]

Faculty of Health and Life Sciences – Wellcome Trust Public Engagement Grants Scheme
End of Project Report

**Title of Project**	
Technology in Palliative Care (TIP) study: public engagement meeting	
**Details of Lead person**	
**Name:**	Dr Amara Nwosu
**Institute:**	Palliative Care Institute Liverpool
**Job title/role:**	Consultant and Honorary Senior Clinical Lecturer in Palliative Medicine
**Email:**	anwosu@liverpool.ac.uk

Q1. Please provide a brief description of what you did in the project including details of who and how many people were involved (include both staff/students and members of the public):

The Wellcome Trust Public Engagement funding was used to conduct a one day workshop on 27th September 2019 at Marie Curie Hospice Liverpool, to discuss and develop research ideas based on the themes from the Technology in Palliative Care (TIP) study. The TIP study aims to identify technology research priorities for palliative care. The TIP study consisted of a scoping review of literature, an International Delphi questionnaire and then a consensus meeting (which took place on 6th September 2019) to agree the technological research priorities for palliative care. A total of 12 priority areas (summarised into 8 themes) were identified from the TIP study.

The TIP public engagement study involved six lay representatives, two staff members (nurse and doctor) and one medical student. The event took place at Marie Curie Hospice Liverpool from 1000 – 1400 and consisted of the following:

- Presentations to introduce the themes and discussion for the day;
- Round table discussions to around the theme areas arising from the TIP study

Agenda:

0930 - 0945 Registration
0945- 1000 Welcome and introductions
1000 - 1020 Why is Technology in Palliative Care important? Dr Amara Nwosu (Consultant and Honorary Senior Clinical Lecturer in Palliative Care, Royal Liverpool University Hospital)
1020 - 1030 Technology in Palliative Care study overview and results. Dr Amara Nwosu (Consultant and Honorary Senior Clinical Lecturer in Palliative Care, Royal Liverpool University Hospital)
1030 - 1115 Group discussion 1
Big Data, Artificial Intelligence, Biotechnology
1115 - 1130 Break
1130 - 1215 Group discussion 2

Telehealth & mobile devices, smart homes, virtual reality, Digital Legacy

1215 - 1245 Feedback and close

1245 - 1400 Lunch

Q2. Please briefly describe the main outputs of the project and the impact the project had on any of the following areas:

Outcomes of the research

Main Outcomes of this research:

- The Technology in Palliative (TIP) Care study has identified 'priority areas' to drive technology focused research in palliative care.
- The TIP study has created an international collaboration of key professional stakeholders to drive research and development of technology in palliative care.

Main outcomes of this public engagement event:

- The Public Engagement event enabled members of the public to share their views on the current and future role of technology in the delivery of palliative care. The group discussed the technology research priorities which were identified from the TIP Delphi study.
- Each priority area was discussed in depth to determine what the public engagement thought about these areas and how they wanted each area to be studied further.

Outcomes for the public involved:

Play an active part in shaping the future research and development into the use of technology in palliative care, specifically:

- This engagement event has formed a Patient and Public Engagement (PPE) group to complement the international collaborative and meaningfully contribute to the ongoing development of research ideas and protocols, to ensure the research is patient centred.
- The public involved had the opportunity to contribute to the development of research ideas, and the design of research projects, into technology in palliative care;
- The attendees of the event were given the opportunity to agree to participate in future work, which will hopefully include roles as a co-applicant for future research projects.

Outcome for staff involved:

Staff and students from the NHS, University and hospice had the opportunity to engage with members of the public to further the research agenda for technology in palliative care.

Outcome for the University of Liverpool

This event has helped the University of Liverpool continue to act as a lead organisation for the developing the research agenda for technology in palliative care, to improve the care of patients with palliative care needs.

Q3. Please describe any anticipated legacy to the project:

This could include things such as the creation of resources that will be used at future events, the development of partnerships that will continue, the up-skilling of students/staff etc.

- Development of public engagement group to participate in future research studies
- Identification of research priorities for technology in palliative care
- Further involvement of Marie Curie Hospice Liverpool in palliative care research.
- Upskilling of hospice-based research nurse
- Involvement of a medical student in a public engagement activity.

Q4. Please give details of any lessons learnt/top tips you would give to someone wanting to do a similar project in the future (this information may be shared on the Faculty website):

Top tips

- Identify a location for your activity and secure a date early
- Consider transportation, mobility issues and dietary needs of public participants carefully.
- Have a plan of how you will advertise your event to your target audience. Consider a variety of different avenues to reach a wider audience if needed (e.g. email, phone, poster, volunteer networks, and charity groups).
- Ensure you have help on the day to help coordinate proceedings (talks, activities, refreshments, registration etc. etc.).
- Ensure you record details of the discussions through the course of the day. If you plan to audi record these discussions, ensure you gain consent from participants beforehand.

Q5. Please provide a brief overview of the project to be published on the Faculty website:
(Maximum 250 words)

The Wellcome Trust Public Engagement funding was used to bring together patient and public representatives at a one day workshop on 27th September 2019 at Marie Curie Hospice Liverpool, to discuss their views on technology-based research priorities for palliative care. This event was linked with the Technology in Palliative Care (TIP) study, which identified 8 priority theme areas through an International Delphi process.

The public engagement event involved six lay representatives, two staff members (nurse and doctor) and one medical student. We facilitated discussion around the priority theme areas, which included: Big Data, Artificial Intelligence, Biotechnology, Telehealth & mobile devices, smart homes, virtual reality and Digital Legacy.

Outcomes from this event include the development of public engagement group to participate in future research studies; identification of research priorities for technology in palliative care; further involvement of Marie Curie Hospice Liverpool in palliative care research; upskilling of hospice-based research nurse and involvement of a medical student in a public engagement activity.

Costing Information

If there were any significant deviations from the original project budget requested please detail them here:

No significant deviation from the original project budget
